# Hospital Meetings

**Published:** 1903-03-21

**Authors:** 


### HOSPITAL MEETINGS.

### EAST LONDON HOSPITAL FOR CHILDREN.

Colonel Needham, who presided over the annual court of this hospital on Thursday, the 26th ult., made an earnest appeal for funds to carry on the work of a hospital of which the very existence, he thought, was unknown to nine-tenths of the inhabitants of the West End. The number of out-patients had trebled in 20 years, but only five beds had been added, and the result was that many who ought to come in had to be treated as out-patients. This showed that another ward was wanted. In spite of the fact that the total receipts reached the sum of £11,000 last year, the financial state of the hospital was hardly satisfactory. £2,000 had to be deducted for the endowment of cots, which reduced the apparent value; that sum had to be invested in perpetuity. Thus the receipts for the general fund remained about the same as in 1901, while there was a considerable falling off in donations. The ordinary expenditure had advanced by £201, accounted for by the necessity of increasing the nursing staff, the hiring of a house for the nurses, and an electrical department. At the same time, economy was systematically practised. The condition attached to the grant of £600 from King Edward's Fund, that necessary improvements should be effected, had been practically observed. A steam laundry, mortuary, and post-mortem room were finished in October. These improvements were necessary, and in spite of the deficit of £664 the committee had the satisfaction of knowing that the work was well done and that it had satisfied the commissioners of King Edward's Fund. The Chairman remarked that he himself had pointed out the urgent need of more accommodation for the nurses and domestic staff, and the Fund had increased their grant by £150. "They think," said Colonel Needham, "that we are inclined to help ourselves, and will encourage us. The grant this year of £1,750 should help us to appeal to the public for further help. We cannot expect help if we do nothing ourselves; we must have modern appliances, and show that the hospital is in line with the efficient hospitals of the Metropolis." The public must be shown that additional beds were urgently needed, so that acute cases might not be turned away. A ward for whooping cough was an absolute necessity, to save the lives of many children who would otherwise die in miserable and unhealthy homes. More out-patients' accommodation was wanted, so that patients might not be kept waiting outside in bad weather. He urged the expediency of individual exertions in making an appeal, and pointed out that those who wore black coats and silk hats could mostly afford five shillings at least.

### ST. MARY'S DAY NURSERY AND HOSPITAL FOR SICK CHILDREN, PLAISTOW.

The annual Court of Governors of that characteristically East End charity the St. Mary's Day Nursery and Hospital for Sick Children, Plaistow, was held at the hospital on Thursday afternoon, the 26th ult. The nurses' dining-room had to be utilised for the meeting, and the remarkable air of cleanliness and freshness prevailing was the more noticeable because of the general character of the surrounding district. The Bishop of Barking (Dr. Stevens) took the chair, and there was a very fair attendance. The usual preliminaries having been transacted, the draft report of the Committee of Management was read by the secretary, Mr. Percy J. Glenton. The committee expressed pleasure on being able to state that the good work carried on by the institution had not diminished during the year, although the number of in-patients received showed a reduction compared with the figures of the two preceding years. But the causes of that reduction were phenomenal, and in no way owing either to weakness in the management or to lack of public support. The report emphasised the urgent need for an additional building for which a site is already secured. The surrounding district is densely populated and this population, which is rapidly increasing, consists entirely of the poorer and more destitute of the working classes. The result is that the limited accommodation of the hospital is taxed to the utmost and the congested state of the waiting-rooms, consulting-rooms, lavatories, and corridors causes difficulties which can only be fully appreciated by those who have to deal with it. No less a sum than £25,000 will be required to provide a hospital capable of meeting the demands of the district, and the committee recognising the absolute necessity of supplying the demand are appealing to the public and have made a good start towards obtaining the amount required—£23,500—a sum of nearly £1,800 having already been raised.

On the motion of Mr. Adamson, seconded by the Rev. T. Given-Wilson, the report and balance sheet were adopted. The balance sheet showed that the cost of maintenance of the hospital had been £3,266, and of the day nursery £58, and that the balance in hand at the beginning of the year of £229 had been reduced to £159.

Six members of the committee of management were then elected and votes of thanks closed the proceedings.

### ST. JOHN'S HOSPITAL FOR DISEASES OF THE SKIN.

The reluctance of governors to attend the business meetings of the institutions with which they are connected presses with peculiar hardship on St. John's Hospital for Diseases of the Skin. For years attempts have been made to alter the rule requiring a quorum of 40 for a special meeting; but a special meeting is requisite to do this, and 40 governors cannot be got to attend for the purpose. Once more on the 27th ult. a Special General Board of Governors was summoned at the Westminster Palace Hotel "to obtain permission to rebuild the hospital on the present site, in Leicester Square, or on some other site as the Board of Management may deem fit," and also to alter the necessary quorum from 40 to 20. Only 39 Governors, however, put in an appearance, and the annual general meeting was, therefore, proceeded with.

Lord Chesterfield presided, and in moving the adoption of the 39th annual report said: I wish to impress on you once again more than ever how necessary it is for us to endeavour to increase, if we possibly can, the list of our annual subscribers. Our report is very short and concise as usual, but its few paragraphs represent a vast amount of work and anxiety on the part of your board and on the part of the medical staff, the nursing staff, and the superintendent, and of all the officials connected with the hospital. With regard to our patients you will notice that the number of in-patients treated during 1902 has been 259, and that was an increase of 20 over 1901. Then you will notice with regard to out-patients the attendances were 7,959 in 1902, as against 7,403 in 1891, which is the large increase of 556. Our income amounted from all sources to £4,066, which is an increase of £95 over that of 1901. The ordinary receipts in 1902 amounted to £3,980 and the extraordinary to £85, compared with £3,883 and £85 in 1901. I should like to point out, and I do it with deep regret, that the subscriptions this year have suffered more than usual from the long list of deaths of those generous supporters of ours who have passed away. In spite of this our ordinary income has more than maintained its level, and your board have every hope that by the vigorous appeal which it is their intention immediately to make, that there will be a considerable increase in these subscriptions, and should like to call your attention for a moment to the paragraph showing that the expenditure for the year was £4,092, as against £4,284 in 1901, which shows a decrease of £192. Comparing the total income with the total expenditure, it will be seen that there was a deficit of £26, as against a deficit of £316 in 1901. I think you will agree that this is a very satisfactory state of affairs, because you will notice that in spite of there having been a considerable increase in the number of our patients our total maintenance and administrative expenses have decreased. This I think will show that every effort has been made to keep down within reasonable bounds the expenses by your executive. Our total indebtedness now amounts to the sum of £872, which is £26 more than last year. This is the skeleton in our cupboard which will continue to stare us in the face until we can collect sufficient funds to give us a surplus of income over expenditure. Passing from the report it is my duty and pleasure to tender our sincere thanks to the medical staff for their services. I cannot speak too highly of the way in which these gentlemen carry out their duties. If I may mention one of these gentlemen in particular—and I do not wish to be invidious—it is Dr. Morgan Dockrell, for he instituted the course of lectures which bears my name, and which, I may venture to say, has been a great success. In fact, whereas in 1901 the average attendance at 26 lectures was somewhere about 40, in 1902 the average attendance was 48 at 27 lectures. I wish further to thank all our honorary officers—the auditors, standing counsel, solicitor, and architect—for the work they have performed on behalf of the hospital this year, and I wish to express my satisfaction also at the way in which our matron and nursing staff have carried out their duties, and have left to the last the mention of our superintendent, Mr. Costine. I regret to inform you that Mr. Costine, after some six and a half years, is about to sever his connection with the hospital. He has obtained another and better post. I feel in parting with Mr. Costine we are parting with a man whose place it will be very hard to fill.

### The Special Hospitals and the King's Fund.

Dr. Morgan Dockrell, in seconding the motion, said that special hospitals were now practically in a state of crisis. They had to deal with the King's Fund. That Fund was originally started by his Majesty to benefit all the hospitals. Unfortunately they found that the advisers of the King's Fund showed a tendency to hand the subscriptions over entirely to general hospitals. In the case of one hospital he knew of, where an application was made to know on what ground a grant was refused, they were told there were three alternatives. The first was to put the shutters up, the second was to rebuild the institution, and the third was to affiliate with a kindred institution. That was all very well, but in a case like their own there was a certain amount of difficulty in doing that, and while they were quite with the King's Fund that the hospital ought to be a large building, they asked the Fund to help them get it. Then it had been urged against them that they had a laboratory, and he was asked by the President of the College of Physicians why they did not send their different sections to some of the recognised institutions, so that these sections could be examined and the diagnoses they made clinically confirmed. He had to demonstrate that the pathologist attached to the ordinary hospital was incapable of diagnosing the different diseases as they presented themselves through the skin, and therefore it was absolutely necessary that they who were the largest skin hospital in the kingdom should have a laboratory with a view to aid their diagnosis. They were treating 1,000 patients a week, and were the one centre for disseminating a knowledge of skin diseases, not only throughout Great Britain, but through all parts of the world. Therefore they deserved encouragement. He might tell them that some of the dermatologists carrying on the special departments of large hospitals came to them to learn.

The Chairman said that Sir Saville Crossley, one of the secretaries of the King's Fund, told him that the reason they did not get a grant, and were not likely to, was owing to the bad state of the building. They had summoned a special meeting to consider the question of rebuilding, and had gone so far as to have plans drawn up, and they wanted about £12,000 to enable them to rebuild on their present site and have the in-patient department with 50 beds again in Leicester Square. They had every reason to believe that they could get rid of the lease of the present in-patient department.

The report was then adopted, and votes of thanks and the election of the Board of Management concluded the proceedings.

### THE GREAT NORTHERN CENTRAL HOSPITAL.

After a preliminary discussion as to whether the report might not be circulated before the date of the meeting, which the chairman promised should be considered, the governors proceeded, on Friday, the 27th ult., to adopt the report and accounts. Sir John Dickson Poynder took the chair, and his announcement of an additional £1,000 a year for the hospital was naturally received with expressions of great satisfaction. The ancient Charity known as the Cloudesley Charity has recently been revised, and under the new scheme the Hospital becomes an ex-officio trustee. A sum of £1,847 in Consols is set aside for the purpose of founding or endowing a convalescent home in connection with the hospital, and this must be established within five years of the date of the scheme. Out of the surplus income of the charity funds a share (which will eventually amount to at least £1,000 a year) becomes payable to the Hospital for general purposes, conditionally upon one of the wards being named the "Richard Cloudesley Ward." This the chairman explained to the assembled governors, and added that great gratitude was due to the Borough Council of Islington, who, through the town clerk, Mr. W. F. Dewey, so energetically represented the interests of the hospital, and with the sympathy and approval of the original trustees of the Cloudesley Charity had secured for it such valuable recognition. The lack of a convalescent home had frequently been deplored by the hospital committee: it would be not only a boon to the patients, but indirectly a financial help to the hospital. Speaking of finance generally, the chairman said the thanks of the governors were also due to the Borough Council for kindly allowing a Hospital Fund to be incorporated with the Coronation celebrations, whereby the very substantial sum of £1,623 16s. 5d. was realised, and owing to this Fund the proposed festival dinner did not take place. Important sanitary improvements had been carried out, the drainage having been repaired under the supervision of the London Sanitary Protection Association, who not only most generously gave their services, but had agreed to make a yearly inspection of the system free of charge. The great district round the hospital continued growing year by year, and the number of patients consequently increased. The time had now arrived when additions to the accommodation must be considered, more especially for the staff. The plan of temporary buildings on the roof, devised by the committee, was not accepted by the County Council, and he thought rightly so. The committee were rather afraid to extend, nevertheless, they felt that the question must be faced. It was right that the staff, as well as the patients, should have comfortable quarters. Electric light ought to be substituted for gas also, and he was going to make a strenuous effort, with the committee, to introduce these necessary improvements. These developments involved responsibility upon those who undertook them, and they would all cost money. He hoped those who had already done so much would renew their efforts.

Mr. Dewey spoke of the value of the hospital treatment to the employees of the Borough Council; some 70 or 80 men had been inmates during the past few years. This was an Islington Hospital; 80 per cent. of the patients came from that district, and he regretted that Islington did so little in return.

The representative of the Hospital Saturday Fund (Mr Smyth) said he thought even more than nurses' quarters, a children's ward should be added; there were recently 27 children in the general wards of the hospital, and "crying babies" were not conducive to the comfort of grown-up patients.

### WEST END HOSPITAL.

The 24th annual meeting of the West End Hospital was held on Friday last, 27th ult., Sir Lennox Napier presiding.

In moving the report and accounts to December, 1902, he said they could see that, so far as expenditure and receipts went, they had got on very well, and thanks to the efforts of their treasurer they had nearly £500 in the bank. They had been aided from other sources besides subscriptions, donations and legacies. The King's Fund gave them £250, and the Sunday and Saturday Funds gave them £350. With regard to the patients, naturally owing to the building operations the number of in-patients was not up to last year, being only 177. That was owing to the top ward being, so to speak, put out of action in consequence of the erection of the new operating-room. That, he was happy to say, was now finished. The operating-room had had to be supplied with various details which were apparently somewhat difficult to get, and that prevented the building being finished as soon as they expected. The top ward was now being put in order and would very shortly be in full swing again, and able to accommodate the full number of patients. Now they had the new operating-room they would be able to deal with their patients more as a hospital of that kind ought to. The number of out-patients had been a little over 4,000, while the number of cases dealt with by massage and electric treatment had also gone up somewhat. Electrical treatment was of course their great feature. They were proposing now to build an isolation ward. No sooner did they get into full swing than a child developed measles or something of the sort which caused the ward to be closed. Children came in from all districts and no amount of inquiry would prevent occasional cases of infection coming in undetected. They had recently acquired a house in Bryanston Square for the nurses. That would enable them to increase their staff and to house them much better than when they resided on the premises. Nurses, above all people, should be in as healthy a condition as possible, and that could not be expected if they had to live in close proximity to their patients and had somewhat limited sleeping space. The Chairman concluded by warm tributes of praise to the treasurer, the medical staff—who, he said, were practical men as well as professional men, and a great help and guide to the lay committee—the secretary and the matron.

Mr. Willock seconded the motion which was adopted, as was also the medical report presented by Dr. T. Outterson Wood. The retiring members of the committee and the auditors were re-elected, and votes of thanks closed the proceedings.

### NEW HOSPITAL FOR WOMEN.

The annual meeting, treated as a social function, with tea and inspection of the wards, which were daintily decked with flowers, took place at this hospital on Tuesday, March 3rd. The Bishop of London was in the chair, supported by Dr. Clifford Allbut, the Rev. J. Llewellyn Davies, the Rev. H. L. Paget, the Bishop-Designate of Exeter, Mr. A. G. Pollock, Mrs. Scharlieb, Mrs. Stanley Boyd, Miss Cock, and others.

The Chairman made a strong appeal for funds to support the hospital which, he said, was increasing in scope and popularity. That her Majesty Queen Alexandra had consented to give it her patronage was, he thought, a great encouragement. The scheme of endowing a bed in memory of the Emperor and Empress Frederick was an excellent one, and deserved support. In spite of encouragements, funds were urgently needed; indeed, he had never known a hospital that did not want money. The grant of £500 from King Edward's Hospital Fund showed that the Council, of which he was a member, thoroughly approved of the working. He hoped the deficit of £500 would be met by those who believed in the principles on which the hospital was established.

Dr. Clifford Allbut said he was greatly struck with the statistics given in the medical report, which stated that out of 14 most difficult operations, the entire number of patients recovered. That was an unusually fortunate result that could not always be expected. It pointed to this, that disease, to be scientifically treated, must be taken at an early stage. That was what happened here. This was not a gynæcological hospital; its peculiarity consisted in this, that cases about which patients were naturally reticent were brought here in an early stage. The public was not educated up to the absolute necessity for this; and having special opportunities, he wanted the new hospital to pay every attention to this fact in its pathological department. Lay governors were too apt to lose sight of the fact that study and investigation were an integral part of an institution for the treatment of disease. A hospital that lacked such a department was bound to fall behind. He therefore most earnestly appealed for funds to carry on the work of the pathological department, which was only endowed to the extent of £5 during 1902.

The new hospital was young and enthusiastic, and unless some far-seeing and intelligent friends gave money for a scientific department very different results from the remarkable ones he had quoted must be looked for. Studentships of £300 or £400 a year ought to be founded; he believed the brains were ready and waiting, but they could not work without the assurance of a bare living. Speaking generally, he thought it would be a very wholesome thing that there should be a greater degree of *rapprochement* between medical men and women; and he believed this would eventually take place. He thought the time had come when women would be admitted to the great medical and surgical guilds. The matter ought to be kept in view. At the same time he thought there would always be a place for a hospital of this kind, where disease might be caught early, and prevented or at least intercepted. On these grounds he very strongly urged the claims of the hospital.

### WEST HAM AND EAST LONDON HOSPITAL.

THE annual general meeting of the West Ham Hospital took place on March 11th, Mr. T. A. Cook presiding. The chief business of the evening was the election of the committee of management, which was rendered as public as possible by the distribution of voting papers amongst all the governors present, who then struck out the names of those whom they did not wish to nominate for the coming year.

The report was taken as read, but the chairman dwelt briefly upon a few of the most important points. The subscriptions for the past year had increased by £179, but the donations had diminished by £350. The School Children's Hospital Fund amounted to £100 more, while the sum contributed by the festival dinner was larger by £1,500: this was owing almost entirely to the munificent gift of £1,200 by Mr. J. R. Roberts, who desired to wipe out the debt on the hospital and so enable it to start the year "with a clean slate." The hospital had received the splendid donation of £1,400 from King Edward's Hospital Fund towards the extension scheme. They had also received a sum of £950 from the Corporation of West Ham on account of the widening of West Ham Lane. On the other hand there had been a slight increase in expenditure on most items: this was owing to their having had more money to spend. The year before they had been obliged by lack of funds to leave many unnecessary things undone. The nursing expenses had gone up, as they had felt they ought to encourage the probationers to stop on by increasing their remuneration, and they had also endeavoured to obtain as high a class of nurses as possible by the same means. Mr. Cook assured the meeting that the hospital possessed a splendid staff of nurses. On the whole the hospital stood in a much more satisfactory position than it had done at the end of 1901. The chairman then made an earnest appeal on behalf of the Adult Samaritan Fund, by means of which patients had been sent to convalescent homes with the most beneficial results. The committee were negotiating for the purchase of the High School, which was situated at the back of the hospital, and which was shortly to be removed; and they hoped by the next Annual Meeting to have arrived at a decision on the subject. The acquisition of these premises would be of the greatest benefit to the hospital, as they would gain two excellent wards with very little expenditure. There was also a spacious drill shed in capital repair, and a fine piece of ground at the back. When once the extension scheme was completed the hospital would be obliged to spend a large sum of money on fitting up an operating theatre in order to enable the hospital to be up to date in every particular.

### NATIONAL HOSPITAL FOR DISEASES OF THE HEART AND PARALYSIS.

THE annual meeting of this hospital took place on Thursday, the 12th inst., the president being the chairman of the hospital, the Earl of Verulam. The report was read by the secretary, and, with the audited balance-sheet of receipts and disbursements (which was not presented, though the report gave the main details), was unanimously adopted. Lord Verulam said that in consequence of the great strain caused by the war all the hospitals of the country were in difficulties at the present moment, and when it came to a question of who should receive a gift, the large hospitals came first, while the small ones went to the wall. There was, however, a kind providence over them, and he was extremely happy to say that a legacy of £1,000 was to be received which would enable the hospital to meet its liabilities. The large increase of patients (16,195 attendances of out-patients and 154 admissions, an increase of 3,007 and 61 respectively) was a matter for great congratulation. There had been a change in the matron: Miss Ashley had left, and Miss E. Barber had taken her place. He wished to bear testimony to the work of the medical and nursing staff, which was beyond praise. The lot of the patients had been exceedingly lightened by the visits of some of the ladies and gentlemen interested in the hospital. He was quite sure that as long as the hospital continued to do the good it had done in the past it would merit the support it had always received.

